# Development and Validation of the Short Form (JAEN-10) of the Joint Assessment of Equilibrium and Neuromotor Status Scale (JAEN-20)

**DOI:** 10.3390/jfmk9040223

**Published:** 2024-11-06

**Authors:** Ana Belén Peinado-Rubia, María Catalina Osuna-Pérez, David Núñez-Fuentes, Daniel Rodríguez-Almagro, Noelia Zagalaz-Anula, Rafael Lomas-Vega

**Affiliations:** 1 Asociación de Fibromialgia de Jaén (AFIXA), 23008 Jaén, Spain; abpr0003@red.ujaen.es; 2 Department of Health Sciences, University of Jaén, 23071 Jaén, Spain; mcosuna@ujaen.es (M.C.O.-P.); rlomas@ujaen.es (R.L.-V.); 3 Residencia Mayores Reifs. Cazalilla, 23628 Jaén, Spain; davidnf15@gmail.com; 4 Department of Nursing, Physiotherapy and Medicine, University of Almería, La Cañada de San Urbano, 04120 Almería, Spain; dra243@ual.es

**Keywords:** balance control, chronic fatigue syndrome, disability evaluation, dizziness, fibromyalgia

## Abstract

**Objectives:** The objective of this study was to develop and validate the short version of The Joint Assessment of Equilibrium and Neuromotor Status Scale (JAEN scale) for use in women with Fibromyalgia Syndrome (FMS) to make the balance disorder measurement process more efficient. **Methods**: A cross-sectional observational validation study was conducted. Fifty-six women with FMS and forty-four healthy controls were included. Certain items from the original tool were selected with the aim of (1) improving internal consistency by reducing item redundancy and (2) obtaining a diagnostic capacity with an area under the ROC curve (AUC) greater than 0.70 for discriminating FMS patients and fallers. The internal consistency, factorial validity, concurrent validity and diagnostic capacity of the new tool were analyzed. **Results**: Factorial analysis showed a two-factor structure that explained 72% of the variance. Cronbach alpha coefficients of 0.904 were obtained for the total score of the JAEN-10 items. Concurrent validity analysis showed strong correlations of the JAEN-10 with other instruments that measured quality of life, postural balance or disability related to dizziness. The score of the JAEN-10 items showed an AUC of 0.858 with a sensitivity of 64.29 and a specificity of 95.45 for discriminating between FMS and healthy controls, and an AUC of 0.835 with a sensitivity of 90.48 and a specificity of 67.24 for discriminating between fallers and non-fallers. **Conclusions**: The 10-item JAEN scale is a valid instrument for discriminating between subjects with or without FMS and between fallers and non-fallers. Its psychometric properties are good and are similar to those of the original 20-item scale. Moreover, it is quicker to complete, which may be relevant for subjects with a tendency to experience fatigue.

## 1. Introduction 

Balance disturbance is common in patients with Fibromyalgia Syndrome (FMS); loss of balance is classified as one of the top ten most disabling symptoms of this syndrome, with a reported prevalence of 45% [1]. Recent studies have highlighted that FMS patients have an increased risk of losing their balance and falling compared to healthy individuals, demonstrating a Fall Risk Index (FRI) of 45% [2]. Several studies have identified an increased prevalence of falls in this population [3,4], while others have pointed to an alteration in sensory integration of visuo-vestibular information as a possible cause of balance disorder in FMS patients [5,6]. Symptoms such as pain and chronic fatigue seem to have significant repercussions for proprioception in these patients, causing greater deterioration of their somatosensory system [7]. Although some studies have found exercise to have a moderate effect on improving balance in subjects with FMS [8,9], only vestibular rehabilitation has shown signs of effectively preventing falls [10]. Nevertheless, knowledge of the effectiveness of this therapy is still scarce.

Postural balance in subjects with FMS is evaluated with the same procedures used in other adult patients; these include posturography, subjective measurements and functional scales focused on objective evaluations. For the measurement of balance in patients with FMS, other tools are classically available, such as the Berg Balance Scale (BBS) [11,12,13] or the BESTest [3], although these tools have been rarely used in this type of patient, possibly because their difficult application makes them less suitable for a population prone to fatigue. Recently, the “Joint Assessment of Equilibrium and Neuromotor Status” (JAEN scale) instrument was created to analyze postural balance in patients with FMS both quantitatively and qualitatively [14]. This scale is made up of 20 tests that rate performance using scores from “0” (normal functioning) to “4” (total limitation). The scale provides a total score ranging from 0 to 80 points and the higher the score, the greater the dysfunction. It should be noted that four subscale scores can also be extracted. The scale showed good psychometric properties, although the Cronbach’s alpha value could indicate redundancy in the items. Furthermore, the scale has excellent sensitivity to change after treatment with vestibular rehabilitation [10] and is accepted as a good, functional evaluation instrument for FMS [15].

Although the validation article of the Joint Assessment of Equilibrium and Neuromotor Status Scale (JAEN scale) indicated that it takes 12–13 min to complete, it could nonetheless induce fatigue in both patients and evaluators. Moreover, some of the test items could be redundant. In light of these factors, reducing the number of items on the scale would make the tool more user-friendly and efficient when measuring balance disorder and the risk of falls in subjects with FMS [14].

Therefore, the aim of this study was to develop and validate a short form of the JAEN scale that makes the process of measuring balance disorder more efficient while maintaining its effectiveness. 

## 2. Materials and Methods

### 2.1. Study and Sample

To meet the objectives of this research, a cross-sectional observational validation study was conducted. The study was carried out in compliance with all legal regulations and the principles of the Declaration of Helsinki. In addition, the protocol was approved by the Research Ethics Committee of the University of Jaen (protocol number JUL.23/6 PRY).

The study subjects belonged to the Jaén Fibromyalgia Association (AFIXA). To be included in the group of patients with FMS, the subjects had to meet the following eligibility criteria: (1) to be a woman (2) to be >18 years old and (3) to fulfil all the diagnostic criteria for FMS, as described in the 2016 American College of Rheumatology (ACR) study. Healthy controls were recruited through advertisements on different social networks and had to fulfil the following inclusion criteria: (1) to be a woman, (2) to be >18 years old and (3) to not fulfil the diagnostic criteria for FMS. The final sample included 100 women, of whom 56 had FMS and 44 were controls who did not have FMS.

To calculate the sample size, the criterion of admitting a minimum of 80 subjects with a total of 10 subjects per item on the scale was followed [16].

### 2.2. Measurements

The basic sociodemographic and anthropometric variables collected were age, weight and height, from which the body mass index (BMI) was calculated.

To assess the symptom severity, discomfort and functional capacity of people with FMS, the Spanish version of the Fibromyalgia Impact Questionnaire (FIQ) was used. This FIQ assesses pain, rigidity, fatigue, depression and anxiety, disability and general well-being related to FMS. It scores from 0 to 100, with 100 representing the maximum negative impact of the disease on the patient [17].

The Spanish version of the SF-12 questionnaire was used to measure the health status of the subjects [18]. This tool is a self-administered questionnaire that was extracted from the SF-36 using multiple regression. The SF-12 questionnaire contains twelve items from which two scores can be extracted: the Physical Component Summary (PCS) and the Mental Component Summary (MCS).

The Spanish version of the Dizziness Handicap Inventory (DHI) was used to assess disability due to dizziness [19]. This is a self-administered questionnaire comprising 25 questions that provide a total score from 0 to 100 points. The higher the score, the greater the disability due to dizziness. In addition to the total score, the emotional, functional and physical subscales can be extracted. The Spanish version of the DHI presents good psychometric properties (α = 0.87) [19]. 

In this study, balance confidence was measured with the Spanish version of the activity-specific balance confidence scale (ABC-16) [20]. This questionnaire contains 16 items that can be scored from 0 to 100 depending on the degree of confidence in balance. The total score is obtained by averaging the results of all the items and can range from 0 to 100, where 100 implies total confidence in the balance. The Spanish version of the ABC was validated with vestibular patients. It demonstrated excellent internal consistency and a possible three-dimensional structure.

The ability to avoid falls was measured with the Spanish version of the Falls Efficacy Scale-International (FES-I) [21]. The FES-I is a questionnaire containing 16 items that measure fear of falling during social and physical activities inside and outside of the home. A higher score on the FES-I is associated with a greater fear of falling. The Spanish version of the FES-I presents acceptable psychometric properties with high internal consistency and a unifactorial structure with two underlying dimensions related to physical activities of different demands.

The number of falls in the last 12 months was recorded by asking participants to answer the following question: “How many falls have you had in the last year?”. A fall was defined as ‘‘an unexpected event in which the participants come to rest on the ground, floor, or lower level [22]’’. A subject was considered to be a faller when they had at least one fall during the study period [23]. Based on this variable, the subjects were classified as fallers or non-fallers.

The JAEN scale [17] is composed of 20 items derived from classic balance evaluation tests. However, it scores on a scale from 0 to 4, where “0” indicates no problem and “4” indicates a complete problem that prevents the patient from performing the actions detailed in the items. The total score ranges from 0 to 80 points, with a higher score indicating a more serious balance disturbance. The tool has shown a good ability to discriminate subjects both at risk of suffering falls and those with the balance impairment associated with FMS. However, the analysis of the items showed a high internal consistency with an alpha value that could indicate item redundancy.

### 2.3. Items Reduction Procedure

The study focused on reducing the number of items in the original version to shorten the time required to complete it. This was achieved by eliminating redundant items detected in the original validation study while maintaining the effectiveness of the tool. The extraction of items from all subscales of the original tool followed the following steps:

1. An analysis of differences in means was performed to explore the ability of each item to differentiate between subjects with and without FMS. The criterion of obtaining a *p* value < 0.05 in the non-parametric Mann–Whitney U test was adopted.

2. In the second step, the same analysis was conducted to prove that the items could differentiate between fallers and non-fallers. To be pre-selected, an item had to fulfil both criteria.

3. In the analysis conducted for each subscale, items that contributed to maintaining the scale’s Cronbach’s alpha close to the optimal value of 0.90 were retained.

4. Finally, a factor analysis was performed to select items that loaded (could be included) clearly on any of the resulting factors.

### 2.4. Statistical Analysis

The data were described using means and standard deviations or frequencies and percentages for continuous or categorical variables, respectively. The exploration of differences in item scores between subjects and healthy controls and between fallers and non-fallers was carried out with the non-parametric Mann–Whitney U test.

Cronbach’s α coefficient was used in the internal consistency analysis. This coefficient evaluates the extent to which the items of an instrument are correlated [24]. An α < 0.70, between 0.70 and 0.90 or >0.90, indicates low internal consistency, good internal consistency or the presence of redundancy, respectively [25].

Construct validity was evaluated using a principal component analysis with Varimax rotation. To check whether the sample was suitable for factor analysis, the Kaiser–Meyer–Olkin (KMO) test was used. Principal component analysis is a multivariate statistical tool that is used to reduce the dimensions of a large dataset with maximum information in the body of data into a few principal components [26].

Pearson’s r coefficient was used to analyze the concurrent validity of the JAEN scale with the number of falls in the last 12 months, DHI and its subscales, ABC-16 and FES-I as well as with the PCS and MCS of the SF-12. An r coefficient < 0.3 is considered insignificant, an r coefficient between 0.3 and 0.5 is considered moderate and an r coefficient > 0.5 is considered strong [27].

To analyze the diagnostic validity of the JAEN scale for discriminating between patients and healthy controls, and between fallers and non-fallers, a receiver operating characteristic (ROC) curve analysis was carried out [28]. The area under the ROC curve (AUC) was used to analyze the ability of the JAEN scale to discriminate between patients and controls. The precision of an instrument in discriminating between patients and controls is considered null if the AUC = 0.5, low between 0.5 and 0.7, good between 0.7 and 0.9 and high for an AUC > 0.9 [29]. Through the analysis of ROC curves, the optimal cut-off points of the JAEN scale score were obtained, which determined the threshold from which the predictive values could be calculated. Sensitivity was defined as the proportion of subjects with FMS (or fallers) who tested positive on the JAEN scale. Following this line of reasoning, specificity was calculated as the proportion of controls (or non-fallers) who had negative results on the JAEN scale. The positive predictive value (PPV) was defined as the proportion of subjects with a positive JAEN score who had FMS (or had fallen). The negative predictive value (NPV) was defined as the proportion of patients with a low JAEN score who were healthy controls (or non-fallers).

Data management and analysis were performed using the Statistical Package for Social Sciences (SPSS) version 27 (SPSS Inc., Chicago, IL, USA) and MedCalc^®^ Statistical Software version 22.023 (MedCalc Software Ltd., Ostend, Belgium; https://www.medcalc.org; 6 August 2024). We worked with a confidence level of 95%, that is, an alpha error of 5% was admitted (*p* < 0.05).

## 3. Results

**Figure 1:**
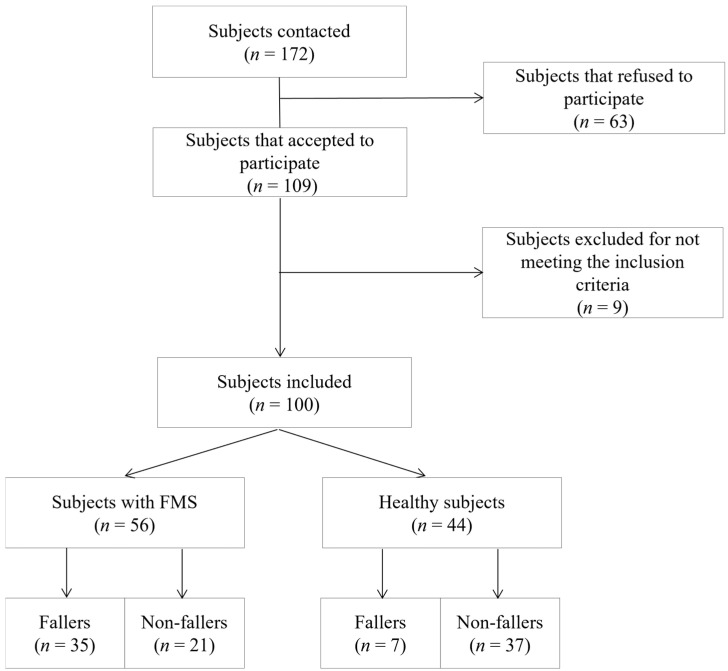
Flow diagram of the participants.

A total of 100 women were selected and agreed to participate in the study. Of these, 56 met the criteria for FMS and 44 were healthy controls (Figure 1). The morphological characteristics and balance impairment levels are shown in Table 1. In general, there were no significant differences between the groups in the morphological variables, although weight difference and the average number of falls in the last year were at the limit of statistical significance in favour of women with FMS. Regarding quality of life, disability due to dizziness and all of the secondary variables, the sample of women with FMS had worse health status with very significant differences.

After the item selection criteria were applied, a total of 10 items were pre-selected (1, 7, 9, 10–14, 19–20). The selected items are shown in Table 2. These items represent the subscales of the original tool and their scores were statistically different between subjects with FMS and controls, as well as between fallers and non-fallers.

The internal consistency analysis of the new scale provided an alpha value of 0.904, which represents a balance between having the best internal consistency and no redundant items. The analysis of the items (Table 2) showed that the elimination of items 2 and 3 could increase the alpha value, although the change was discrete and, in any case, would increase the indicator of item redundancy [25].

Regarding the principal component analysis, the Kaiser–Meyer–Olkin (KMO) measure was 0.849 and the Bartlett sphericity test was equal to 836.769 (*p* < 0.001). Therefore, the sample can be considered optimal for principal component analysis. This analysis showed a dimensional structure that explained 72% of the variance in two factors (Table 3). Table 4 shows the items included in each of the two factors identified: (1) First Factor = Instability when Support is Reduced with Eyes Closed (SREC): balance related to more demanding sensory conditions (Items 1, 2 and 3); (2) Second Factor = Instability when Standing and Walking with Head Movements (SWHM): static and dynamic balance related to head movements (Items 4–10).

The concurrent validity analysis of the 10-item JAEN showed high correlation of all variables measuring balance (DHI, ABC and FES) and other impact measures of quality of life (FIQ and Physical Component Summary of SF-12), except the Mental Component Summary of the SF-12, for which the correlation was moderate. Additionally, it can be observed that the different correlations with JAEN-20 and JAEN-10 are practically identical (Table 5).

**Figure 2:**
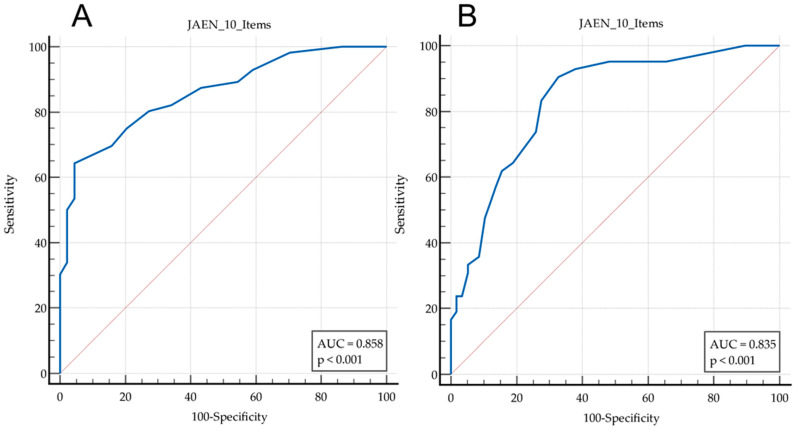
(**A**): ROC curve showing the ability of the JAEN-10 scale to discriminate between patients with or without Fibromyalgia Syndrome; (**B**): ROC curve showing the ability of the JAEN-10 scale to discriminate between fallers and non-fallers.

Regarding diagnostic validity (Table 6), the ROC curve analysis showed that a cut-off point < 14 showed a sensitivity of 64% and a specificity of 95% for the discrimination of subjects with or without FMS (in terms of balance disorders). Regarding falls, a cut-off point < 11 provided a sensitivity of 90% for detecting fallers, with a sensitivity of 67%. The area under the ROC curve (Figure 2) for the discrimination of patients with FMS was 0.858 (0.774 to 0.920, *p* < 0.001), while for fallers it was 0.835 (0.748 to 0.902, *p* < 0.001).

## 4. Discussion

This study aimed to develop and validate a short form of the instrument “Joint Assessment of Equilibrium and Neuromotor Status” (JAEN scale) to improve the evaluation of postural balance and the prediction of fall risk in subjects with FMS [14]. The original instrument (JAEN-20 items) already shows good internal consistency, perhaps even excessively so for the criteria of some authors [25]. It also shows greater sensitivity to change than other commonly used tools. As a result of this study, a 10-item version (JAEN-10 items) has been obtained that can be completed in about 5 min.

In comparison to the original version, the JAEN-20 items presented an internal consistency alpha = 0.928 [14] which may indicate item redundancy. The JAEN-10 items (Supplementary Table S1) scale has an alpha value = 0.904, which implies an optimal value with no redundancy [25]. Regarding construct validity, while the extended version JAEN-20 items provided four subscales, the short version, JAEN-10 items, only has a two-factor structure: Factor 1: Instability when Support is Reduced with Eyes Closed (SREC), and Factor 2: Instability when Standing and Walking with Head Movements (SWHM). While this may appear to be a limitation, it actually makes the short version more suitable for use in clinical settings, as it is easier to interpret. Additionally, although the AUC for the diagnostic prediction of FMS is reduced from 0.928 on the JAEN-20 to 0.858, the tool improves the precision of detecting falling subjects from 0.795 on the JAEN-20 to 0.835 of the JAEN-10 items version. Finally, the concurrent validity of both the standard and short instruments is almost identical to other scales. All the above features, plus the reduction in implementation time by half, make the JAEN-10 version more versatile for use in clinical settings when compared to the extended version (JAEN-20).

One of the main reasons why the authors designed the extended version of the JAEN scale (JAEN-20) was due to how simple it was to complete for subjects prone to fatigue. This made the JAEN-20 items a more convenient tool than the Berg Balance Scale (BBS), the BEStest or the expensive computerized dynamic posturography. While the BBS has sometimes been used to measure balance in FMS [11,12,13], the BESTest was only used once [3]. It should be noted that the concurrent validity of the FIQ with the two versions of the JAEN scale (r = 0.40–0.45) is similar to that of the BBS (r = −0.48) and the BESTest (r = −0.43), showing moderate correlation [3].

However, although the use of JAEN-10 items may be suitable in clinical settings for assessing and monitoring balance disorder progression, the standard version, JAEN-20 items, may be more useful in research settings, as the subscales reveal potential balance impairment specifically due to the alteration of visuo-vestibular afferents. This was also proven with computerized dynamic posturography [5], but at a higher cost given the latest technological developments. The JAEN-20 items would also be appropriate for measuring changes in patients’ health status, as it has shown a certain degree of responsiveness [10]. However, this property is yet to be evaluated for the short version, JAEN-10 items.

In the opinion of the authors, with the information provided in this paper, the JAEN-10 could become the most versatile clinical tool for inclusion both in the initial evaluation of patients with FMS and for monitoring patients’ progress. During the initial evaluation, the JAEN-10 could be used as a screening tool for patients with balance disorders who could benefit from a specific rehabilitation programme. Once the patients have undergone balance rehabilitation, the JAEN-10 could be used to monitor their progress.

This study has numerous limitations. Firstly, the sample only comprised women. However, this is representative of the much higher prevalence of FMS in women than in men. Furthermore, all patients were from the same geographical area, which makes extrapolating our results to other settings difficult. On the other hand, our study did not evaluate intrinsic or extrinsic factors related to falls. In addition, the group of women in the study has been analyzed several times with the JAEN scale during clinical management and other studies, which could affect their performance in the tests due to the practice of performing them, although as it is an objective test, memory bias is not likely. Furthermore, although several psychometric properties have been measured for this new instrument, others are yet to be evaluated, including interobserver reliability and responsiveness. These factors should be evaluated in further research. It would also be desirable to extend its validation to different populations beyond solely subjects with FMS.

## 5. Conclusions

The JAEN-10-item scale is a valid instrument for measuring postural balance and risk of falls in women with FMS. This tool can be implemented quickly and interpreted relatively easily given the thresholds that may indicate the possible presence of FMS (14 points) and/or risk of falls (11 points). Further research should be conducted to verify interobserver reliability, sensitivity to change and the ability to predict falls prospectively. It should also aim to validate the use of the instrument in different populations.

## Figures and Tables

**Table 1:** Morphologic and clinical variables.

Variables	Healthy	*n* = 44	FMS	*n* = 56	*p*-Value
Statistics	Mean	SD	Mean	SD	*t*-test
Age	61.00	10.69	57.75	7.61	0.082
Height	1.60	0.06	1.60	0.06	0.665
Weight	68.18	10.45	73.70	14.78	0.052
Body Mass Index	26.91	4.35	28.68	5.25	0.093
Years since diagnosis			14.00	9.28	
Falls in the last year	0.55	2.33	1.23	1.25	0.062
Physical Component Summary SF-12	49.35	8.67	30.74	5.81	<0.001
Mental Component Summary SF-12	47.04	9.89	32.86	10.26	<0.001
Dizziness Handicap Inventory Emotional	2.73	5.96	15.11	8.29	<0.001
Dizziness Handicap Inventory Functional	5.09	7.92	18.98	8.88	<0.001
Dizziness Handicap Inventory Physical	7.50	8.25	19.27	5.36	<0.001
Dizziness Handicap Inventory Total Score	15.32	19.38	53.36	20.47	<0.001
Activities Balance Confidence Scale	85.15	13.42	58.19	22.55	<0.001
Falls Efficacy Scale	22.12	6.05	34.46	10.78	<0.001

SD: standard deviation, SF-12: 12-Item Short-Form Health Survey, FMS: Fibromyalgia.

**Table 2:** Items analysis of the short form of the Joint Assessment of Equilibrium and Neuromotor Status Scale (JAEN-10).

	Mean of the Scale If the Element Is Deleted	Scale Variance If the Element Is Removed	Corrected Total Element Correlation	Multiple Squared Correlation	Cronbach’s Alpha If Element Is Deleted
Romberg test	13.08	48.499	0.505	0.341	0.903
One Leg Left (Eyes Closed)	10.80	51.253	0.289	0.470	0.914
One Leg Right (Eyes Closed)	10.76	50.406	0.365	0.478	0.910
Rotational head shaking test (Eyes Closed)	13.31	43.792	0.803	0.773	0.885
Head shaking test on the left-right diagonal (Eyes Open)	13.26	42.699	0.808	0.859	0.884
Head shaking test on the left-right diagonal (Eyes Closed)	13.01	42.050	0.820	0.819	0.883
Head shaking test on the right-left diagonal (Eyes Open)	13.24	43.134	0.820	0.866	0.884
Head shaking test on the right-left diagonal (Eyes Closed)	13.15	42.412	0.853	0.878	0.881
Walk Shaking Neck Flexion (Eyes Open)	13.06	46.097	0.664	0.675	0.894
Walk Shaking Neck Rotation (Eyes Open)	12.69	45.772	0.617	0.649	0.897

**Table 3:** Explained variance of the 10-item Joint Assessment of Equilibrium and Neuro-motor Function (JAEN) screening tool items by principal component analysis.

Component	Initial Eigenvalues			Sums of the Squared Saturations of the Extraction			Sum of the Squared Saturations of the Rotation		
	Total	% of Variance	% Accumulated	Total	% of Variance	% Accumulated	Total	% of Variance	% Accumulated
1	5.552	55.524	55.524	5.552	55.524	55.524	5.162	51.624	51.624
2	1.684	16.839	72.363	1.684	16.839	72.363	2.074	20.739	72.363
3	0.968	9.678	82.040						
4	0.622	6.221	88.262						
5	0.346	3.456	91.718						
6	0.254	2.536	94.254						
7	0.211	2.114	96.368						
8	0.179	1.790	98.158						
9	0.117	1.165	99.323						
10	0.068	0.677	100.000						

**Table 4:** Rotated component matrix of the short form of the Joint Assessment of Equilibrium and Neuromotor Status Scale (JAEN-10) obtained by principal component analysis with Varimax rotation.

	Component	
	1	2
Romberg test		0.578
One Leg Left (Eyes Closed)		0.874
One Leg Right (Eyes Closed)		0.874
Rotational head shaking test (Eyes Closed)	0.893	
Head shaking test on the left-right diagonal (Eyes Open)	0.920	
Head shaking test on the left-right diagonal (Eyes Closed)	0.889	
Head shaking test on the right-left diagonal (Eyes Open)	0.917	
Head shaking test on the right-left diagonal (Eyes Closed)	0.926	
Walk Shaking Neck Flexion (Eyes Open)	0.682	
Walk Shaking Neck Rotation (Eyes Open)	0.636	

**Table 5:** Concurrent validity of the JAEN-20 and JAEN-10 tools with subjective measurement scales of balance disturbance.

	JAEN 10-ITEMS		JAEN 20-ITEMS	
	r Pearson	Correlation	r Pearson	Correlation
Fibromyalgia Impact Questionnaire	0.398	Moderate	0.450	Moderate
Physical Component Summary of SF-12	−0.658	Strong	−0.653	Strong
Mental Component Summary SF-12	−0.441	Moderate	−0.439	Moderate
Dizziness Handicap Inventory emotional	0.599	Strong	0.576	Strong
Dizziness Handicap Inventory functional	0.662	Strong	0.652	Strong
Dizziness Handicap Inventory physical	0.687	Strong	0.704	Strong
Dizziness Handicap Inventory Total Score	0.695	Strong	0.689	Strong
Activities Balance Confidence Scale	−0.542	Strong	−0.607	Strong
Falls Efficacy Scale International	0.532	Strong	0.547	Strong

**Table 6:** Validity of the JAEN-10 items for diagnosing Fibromyalgia Syndrome and falls.

Diagnostic	Criterion	Sen	95% CI	Spe	95% CI	+LR	95% CI	−LR	95% CI	+PV	95% CI	−PV	95% CI
Fibromyalgia	>14	64.29	(50.4–76.6)	95.45	(84.5–99.4)	14.14	(3.60–55.55)	0.37	(0.26–0.53)	94.7	(82.1–98.6)	67.7	(59.5–75.0)
Falls	>11	90.48	(77.4–97.3)	67.24	(53.7–79.0)	2.76	(1.89–4.04)	0.14	(0.055–0.37)	66.7	(57.7–74.5)	90.7	(79.1–96.2)

Sen: sensitivity; 95% CI: 95% confidence interval; Spe: specificity; +LR: positive likelihood ratio; −LR: negative likelihood ratio; +PV: positive predictive value; −PV: negative predictive value.

## Data Availability

The data used to support the findings of this study are available from the corresponding author upon request.

## Supplementary Materials

The following supporting information can be downloaded at https://www.mdpi.com/article/10.3390/jfmk9040223/s1.
