# Supplementary material for: Development and Validation of the Short Form (JAEN-10) of the Joint Assessment of Equilibrium and Neuromotor Status Scale (JAEN-20)
Source: J Funct Morphol Kinesiol. 2024 Nov 6;9(4):223. doi: 10.3390/jfmk9040223 (PMC11587025; doi:10.3390/jfmk9040223)
Supplement: Supplementary file 1 [file jfmk-09-00223-s001.zip › jfmk-3221321-supplementary.pdf]

Table S1: The short form of the Joint Assessment of Equilibrium and Neuromotor Status Scale (JAEN-10).

	DISABILITY SCORE ACCORDING TO THE INTERNATIONAL CLASSIFICATION OF FUNCTIONING, DISABILITY AND HEALTH (ICF)					
	NO PROBLEM =0	MILD PROBLEM=1	MODERATE PROBLEM=2	SEVERE PROBLEM=3	COMPLETE PROBLEM =4	SCORE
STATIC BALANCE TEST						
1.-Standing Eyes Closed (Romberg test)	Straight body position and stable (30 seconds)	Lateral deviation of the body but stable	Instability	Fall / finish before 30 seconds	Fall / finish before 15 seconds	
2.-Standing Tandem Left	Straight body position and stable (30 seconds)	Lateral deviation of the body but stable	Instability	Fall / finish before 30 seconds	Fall / finish before 15 seconds	
3.-Tandem Romberg Left	Straight body position and stable (30 seconds)	Lateral deviation of the body but stable	Instability	Fall / finish before 30 seconds	Fall / finish before 15 seconds	
4.-Standing Tandem Right	Straight body position and stable (30 seconds)	Lateral deviation of the body but stable	Instability	Fall / finish before 30 seconds	Fall / finish before 15 seconds	
5.-Tandem Romberg Right	Straight body position and stable (30 seconds)	Lateral deviation of the body but stable	Instability	Fall / finish before 30 seconds	Fall / finish before 15 seconds	
6.-One-Legged Stance Time Left Eyes Open	Straight body position and stable (30 seconds)	Body stabilizing movements but only of the trunk	Body stabilizing movements including the load foot	Bipodal support before 30 seconds	Bipodal support before 15 seconds	
7.-One-Legged Stance Time Left Eyes Closed	Straight body position and stable (15 seconds)	Body stabilizing movements but only of the trunk	Body stabilizing movements including the load foot	Bipodal support before 15 seconds	Impossibility	
8.-One-Legged Stance Time Right Eyes Open	Straight body position and stable (30 seconds)	Body stabilizing movements but only of the trunk	Body stabilizing movements including the load foot	Bipodal support before 30 seconds	Bipodal support before 15 seconds	
9.-One-Legged Stance Time Right Eyes Closed	Straight body position and stable (15 seconds)	Body stabilizing movements but only of the trunk	Body stabilizing movements including the load foot	Bipodal support before 15 seconds	Impossibility	
10.-Modified Head Shaking Rotation Test Eyes Closed	Straight body position and stable. Without symptoms	Dizziness/ instability at the end. Mild symptoms	Constant Dizziness/ instability. Moderate/severe symptoms	Fall or need to interrupt <30 seconds	Fall or need to interrupt <15 seconds	

Table S1: The short form of the Joint Assessment of Equilibrium and Neuromotor Status Scale (JAEN-10).

**11.-Modified Head Shaking Flexion Test Eyes Open Left (with 45º left rotation)**	**Straight body position and stable. Without symptoms**	**Dizziness/ instability at the end. Mild symptoms**	**Constant Dizziness/ instability. Moderate/severe symptoms**	**Fall or need to interrupt <30 seconds**	**Fall or need to interrupt <15 seconds**	
**12.-Modified Head Shaking Flexion Test Eyes Closed Left (with 45º left rotation)**	**Straight body position and stable. Without symptoms**	**Dizziness/ instability at the end. Mild symptoms**	**Constant Dizziness/ instability. Moderate/severe symptoms**	**Fall or need to interrupt <30 seconds**	**Fall or need to interrupt <15 seconds**	
**13.-Modified Head Shaking Flexion Test Eyes Open Right (with 45º right rotation).**	**Straight body position and stable. Without symptoms**	**Dizziness/ instability at the end. Mild symptoms**	**Constant Dizziness/ instability. Moderate/severe symptoms**	**Fall or need to interrupt <30 seconds**	**Fall or need to interrupt <15 seconds**	
**14.-Modified Head Shaking Flexion Test Eyes Closed Right (with 45º right rotation).**	**Straight body position and stable. Without symptoms**	**Dizziness/ instability at the end. Mild symptoms**	**Constant Dizziness/ instability. Moderate/severe symptoms**	**Fall or need to interrupt <30 seconds**	**Fall or need to interrupt <15 seconds**	
15.-Sphinx Pose during 30 seconds.	Without symptoms	Neck discomfort	Vegetative symptoms	Need to interrupt <30 seconds	Need to interrupt <15 seconds	
16.-Fukuda Stepping Test 50 Step Eyes Closed.	Deviation <30º to either side	Deviation to one side >30º	Get to finish but with ataxia	Fall or need to interrupt <30 seconds	Fall or need to interrupt <15 seconds	
17.-Babinski-Weill test (30 steps)	Straight position and stable	Deviation to one side <30º	Deviation to one side >30º	Fall or need to interrupt <30 seconds	Fall or need to interrupt <15 seconds	
18.-Walk tandem Eyes Open (30 seconds)	Straight position and stable	Get to finish but with instability/ dizziness	Get to finish but with ataxia	Fall or need to interrupt <30 seconds	Fall or need to interrupt <15 seconds	
**19.-Walk Shaking Neck Flexion-Extension Eyes Open (30 seconds)**	**Straight position and stable**	**Get to finish but with instability/ dizziness. Decrease step frequency**	**Get to finish but with ataxia**	**Fall or need to interrupt <30 seconds**	**Fall or need to interrupt <15 seconds**	
**20.-Walk Shaking Neck Rotation Eyes Open (30 seconds)**	**Straight position and stable**	**Get to finish but with instability/ dizziness. Decrease step frequency**	**Get to finish but with ataxia**	**Fall or need to interrupt <30 seconds**	**Fall or need to interrupt <15 seconds**	
**TOTAL SCORE**						
